# Expanding the Genotypic and Phenotypic Spectrum of SPENCDI: A Novel *ACP5* Variant and Literature Review

**DOI:** 10.3390/genes17040390

**Published:** 2026-03-29

**Authors:** Wei Li, Jinrong Li, Decheng Jiang, Xiao Fu, Ping Li

**Affiliations:** 1 Department of Pediatrics, West China Second University Hospital, Sichuan University, Chengdu 610041, China; liweisehuna@163.com (W.L.); lijinrong224@163.com (J.L.); fuxiao12369@163.com (X.F.); 2 Key Laboratory of Birth Defects and Related Diseases of Women and Children (Sichuan University), Ministry of Education, Chengdu 610041, China; 3 Department of General Surgery, Peking Union Medical College Hospital, Peking Union Medical College, Chinese Academy of Medical Sciences, Beijing 100023, China; jiang_decheng@126.com; 4 Key Laboratory of Research in Pancreatic Tumor, Chinese Academy of Medical Sciences, Beijing 100023, China; 5 National Science and Technology Key Infrastructure on Translational Medicine in Peking Union Medical College Hospital, Beijing 100023, China

**Keywords:** autoimmunity, immunodeficiency, spondyloenchondrodysplasia, skeletal dysplasia, *ACP5*

## Abstract

Introduction: Spondyloenchondrodysplasia with immune dysregulation (SPENCDI) is a rare autosomal recessive disorder caused by biallelic variants in the *tartrate-resistant acid phosphatase 5* (*ACP5)* and characterized by variable skeletal, immunological, and neurological manifestations. Because early skeletal abnormalities may be subtle, diagnosis can be challenging in infancy. Materials and methods: We conducted a detailed clinical, immunological, radiological, and molecular evaluation of an infant with early-onset cytopenia, recurrent infections, seizures, and developmental delay. Genomic analysis was performed using whole exome sequencing (WES) and copy number variation sequencing (CNV-seq). In addition, we performed a structured narrative review of published *ACP5*-related SPENCDI cases to summarize the clinical spectrum and the currently reported use of Janus kinase (JAK) inhibitors. Results: Genomic analysis identified an *ACP5* stop-gain variant (c.311G>A; p.Trp104*) with an apparently homozygous signal on WES. Re-evaluation of the copy-number data demonstrated an overlapping heterozygous 19p13.2–p13.13 deletion encompassing *ACP5*, indicating biallelic *ACP5* defects consisting of a sequence variant on one allele and deletion of the other allele. Clinically, the patient showed prominent extra-osseous manifestations, including impaired T- and NK-cell cytotoxicity, before the emergence of definite radiographic skeletal abnormalities. Our literature review showed that skeletal abnormalities were repeatedly documented across published *ACP5*-related SPENCDI reports, although radiographic changes were often subtle and could be preceded by immune manifestations. Reported use of JAK inhibitors suggests potential benefit for immune dysregulation in selected patients, whereas the neurological response remains uncertain. Conclusions: This study reports a novel *ACP5* variant and expands the known phenotypic spectrum of SPENCDI. SPENCDI should be considered in children with unexplained immune dysfunction and developmental delay, and suggestive neuroimaging findings, even when overt skeletal deformities are absent. Early genetic testing and targeted skeletal imaging may facilitate diagnosis.

## 1. Introduction

Spondyloenchondrodysplasia with immune dysregulation (SPENCDI) is a rare congenital skeletal dysplasia characterized by spondyloepiphyseal dysplasia and enchondromatous-like changes in bone [1]. SPENCDI results from autosomal recessive inheritance of biallelic variants in the *tartrate-resistant acid phosphatase 5* (*ACP5)*. *ACP5*, located on chromosome 19p13.2, may harbor missense, nonsense, frameshift, and splice-site variants [2]. It encodes tartrate-resistant acid phosphatase (TRAP) [3], an enzyme involved in bone resorption and immune regulation [4,5]. Loss of *ACP5* function has been linked to abnormal bone remodeling and dysregulation of the immune system including aberrant type I interferon signaling [2,4].

SPENCDI predominantly affects children and may involve both immune dysregulation and immunodeficiency. Immune dysregulation can manifest as thrombocytopenia, autoimmune hemolytic anemia, systemic lupus erythematosus (SLE), and other autoimmune disorders [6]. Immunodeficiency may present with recurrent bacterial or viral infections associated with reduced B-, T-, or NK-cell numbers or function [7]. Immune dysregulation not only affects the immune system but may also induce neurological dysfunction through abnormally activated immune pathways. Neurological dysfunction includes cerebral calcifications, cerebral atrophy, spasticity, seizures and developmental delay [8]. The features and severity of SPENCDI are polymorphic and associated with significant childhood morbidity and mortality [9]. The clinical management of SPENCDI requires a multidisciplinary approach involving rheumatologists, endocrinologists, immunologists, neurologists, and orthopedic specialists. Current treatments focus on controlling autoimmune symptoms, improving bone health, and managing neurological complications, with potential new therapies being explored. Identifying all genetic variants responsible for SPENCDI is therefore crucial to facilitate the development of targeted treatments.

Here, we describe an infant with *ACP5*-related SPENCDI who presented with early-onset bicytopenia, recurrent infections, seizures, and developmental delay, with skeletal abnormalities becoming apparent only after targeted radiographic evaluation. In addition, we performed a structured narrative review of published *ACP5*-related SPENCDI cases to summarize the clinical spectrum and the currently reported experience with Janus kinase inhibitor therapy.

## 2. Materials and Methods

### 2.1. Ethics Compliance

The research protocol was approved by the Ethics Committee of West China Second Hospital of Sichuan University (Approval No. 2022084). Informed consent for copy number variation sequencing (CNV-seq), whole-exome sequencing (WES), and publication of the patient’s data was obtained from the patient’s legal guardian.

### 2.2. Clinical Chemistry and Imaging

All laboratory analyses were conducted at the Clinical Laboratory Center of West China Second University Hospital. Complete blood count (CBC) testing was performed using automated hematology analyzers (Beckman Coulter systems), with red blood cell (RBC) count and hemoglobin (HGB) quantified via sodium lauryl sulfate (SLS) hemoglobin method, platelet (PLT) count measured by direct current (DC) detection, and white blood cell (WBC) enumeration confirmed via flow cytometry with fluorescent staining. Autoantibody screening, including antinuclear antibody (ANA) and anti-dsDNA antibody detection, utilized indirect immunofluorescence (IIF) with HEp-2 cell substrates, supplemented by chemiluminescence assays for specific antibodies. The direct antiglobulin test (DAT) employed microcolumn gel card assays. Bone marrow aspiration was performed after obtaining informed consent from the patient’s guardians, adhering to sterile protocols with Wright–Giemsa staining for morphological evaluation and flow cytometry (BD FACSCanto™ II) to assess cellularity and exclude malignancies. Cellular immune function profiling included flow cytometric quantification of lymphocyte subsets (CD3+, CD8+ T cells, and CD56+/CD16+ NK cells), with functional assays (e.g., cytotoxic activity via CD107a degranulation or granzyme B release) revealing T and NK cell activity. All procedures followed ISO 15189-certified quality control standards [10], and technical specifications are available upon request.

All imaging examinations were performed at the Department of Radiology, West China Second University Hospital, following pediatric-optimized protocols. X-rays of the limbs and spine were acquired using a Canon SONIALVISION G4 digital radiography system, with anteroposterior and lateral projections. Radiation exposure was minimized through low-kilovoltage techniques, automatic exposure control, and lead shielding for non-targeted areas. Brain CT imaging was conducted using a pediatric-adapted protocol (GE Revolution CT) with iterative reconstruction to reduce radiation dose while maintaining diagnostic resolution. Brain MRI was performed on a Siemens Skyra 3.0 Tesla scanner, incorporating axial and sagittal T1-weighted, T2-weighted, and fluid-attenuated inversion recovery (FLAIR) sequences. For uncooperative pediatric patients, mild sedation (oral chloral hydrate) was administered under anesthesiologist supervision to minimize motion artifacts. All images were analyzed using PACS-integrated workstation by board-certified pediatric radiologists, adhering to the ALARA (As Low As Reasonably Achievable) principle and ISO 15189 quality standards [10]. Informed consent was obtained from guardians prior to procedures involving sedation or contrast agents.

### 2.3. Genetic Testing and Data Analysis

At one year of age, peripheral blood samples were obtained from the proband and his parents for genomic DNA analysis. WES was performed using the IDT xGen Exome Research Panel on the Illumina NovaSeq 6000 platform. Reads were aligned to the GRCh38/hg38 reference genome, followed by standard variant calling and annotation. In the proband, the average sequencing depth across target regions was 169.15×, with base coverage rates of 99.73% at ≥1×, 99.63% at ≥10×, 99.46% at ≥20×, 99.03% at ≥30×, and 96.29% at ≥50×. Parental data were used for segregation analysis.

Because the *ACP5* sequence variant appeared homozygous in the proband but was absent in the mother, the copy-number data were re-evaluated to determine whether the deleted interval overlapped *ACP5*. WES suggested a heterozygous 19p13.2–p13.13 deletion involving *ACP5*. CNV-seq was subsequently performed in the proband as an orthogonal assay to confirm the copy-number abnormality. Because the WES and CNV-seq reports were generated using different reference genome builds, their CNV boundaries are reported exactly as provided in the original diagnostic reports.

The *ACP5* sequence variant identified in the proband was validated by Sanger sequencing, and variant classification was described with reference to the 2015 ACMG guidelines and the original diagnostic report. PCR primers flanking the *ACP5* variant were designed based on the reference transcript NM_001111035.3. Amplification was performed in 50 μL reactions under the following conditions: initial denaturation at 95 °C for 5 min; 30 cycles of 95 °C for 30 s, 60 °C for 30 s, and 72 °C for 30 s; followed by a final extension at 72 °C for 10 min. Bidirectional sequencing was conducted on an ABI 3730XL analyzer, and parental samples were analyzed to assess segregation.

### 2.4. Literature Review Methodology

To better contextualize the present case, we performed a structured narrative review of published reports on *ACP5*-related SPENCDI. We searched PubMed, Web of Science, and Embase through March 2026 using combinations of the terms “*ACP5*”, “spondyloenchondrodysplasia”, “SPENCD”, “SPENCDI”, “tartrate-resistant acid phosphatase”, “TRAP”, “Janus kinase inhibitors”, and “JAK inhibitor”.

We included case reports and case series describing patients with molecularly confirmed *ACP5*-related disease and extractable clinical information. Review articles, duplicate publications, and studies without sufficient clinical or molecular details were excluded. Data extracted from eligible reports included genotype, age at presentation, skeletal manifestations, neurological manifestations, growth parameters, immune dysregulation or immunodeficiency, and reported use of JAK inhibitors. Because some publications may include overlapping patients and reporting completeness varied across studies, pooled percentages and an overall unique-patient total were not calculated. The published evidence was therefore summarized narratively rather than synthesized as a formal systematic review or meta-analysis.

## 3. Results

### 3.1. Clinical Features

The proband exhibited immune and neurological manifestations from birth. He presented with thrombocytopenia, anemia, recurrent infections, seizures, and developmental delay (Table 1). He was delivered by cesarean section at 41 weeks of gestation to non-consanguineous parents, with Apgar scores of 8, 9, and 10 at 1, 5, and 10 min, respectively.

Within the first two years of life, he experienced recurrent pneumonia, enteritis, and sepsis caused by pathogens including *Klebsiella pneumoniae*, *Staphylococcus aureus*, and *Streptococcus pneumoniae*. He also had seizures, severe muscle tone abnormalities, and marked developmental delay. Motor delay was characterized by failure to achieve milestones such as head control, rolling over, independent sitting, and standing. In addition, skeletal abnormalities were observed, including a barrel-shaped chest, scoliosis, varus deformities, and dorsiflexion of the first metatarsophalangeal joints in both feet.

### 3.2. Clinical Chemistry, Hematology, and Biopsy Findings

Blood tests revealed anemia and thrombocytopenia with normal white blood cell counts. Autoantibody testing was negative, and direct antiglobulin test was weakly positive. Bone marrow aspiration showed no abnormalities. Immune function testing demonstrated reduced cytotoxic activity of T cells and NK cells, potentially explaining the recurrent infections caused by specific pathogens.

### 3.3. Imaging

**Figure 1:**
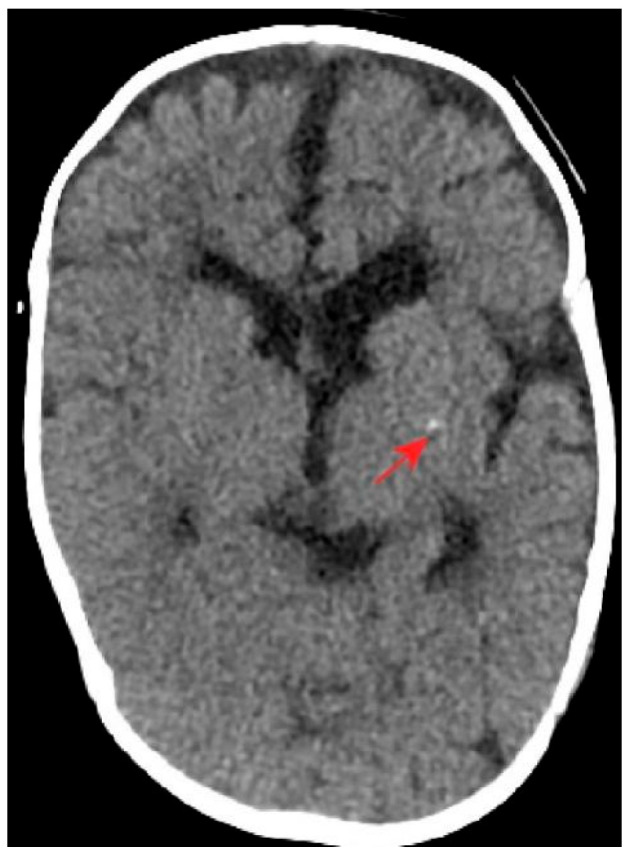
CT imaging of the brain. The arrow indicates basal ganglia calcifications.

**Figure 2:**
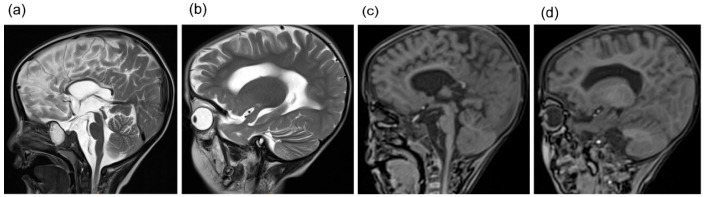
MRI of the brain. (**a**–**d**) Significant thinning of the corpus callosum and structural changes in the anterior horns of the lateral ventricles bilaterally.

**Figure 3:**
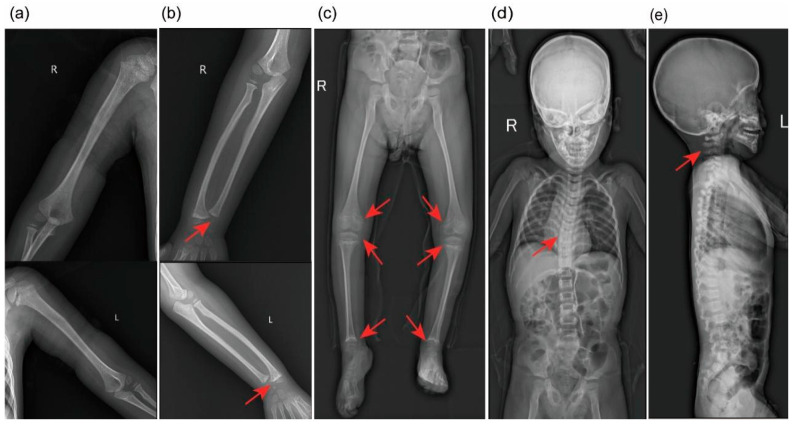
X-rays of the limbs and spine. (**a**–**c**) Metaphyseal lesions of the limbs. (**d**,**e**) Straightening of the cervical spine curvature and thoracic scoliosis.

Computed tomography (CT) of the brain revealed asymmetric cerebral hemispheres, poorly defined right-sided gyri and sulci, a shallow Sylvian fissure, punctate calcifications in the left basal ganglia, widening of the lateral ventricles, and blunting of the left frontal horn (Figure 1). Brain magnetic resonance imaging (MRI) showed marked thinning of the corpus callosum and structural abnormalities involving in the anterior horns of the lateral ventricles bilaterally (Figure 2). Electroencephalography demonstrated frequent polymorphic and sharp slow waves in the frontal, central, occipital, anterior temporal, and posterior temporal regions. X-rays of the limbs and spine revealed irregular metaphyseal morphology and uneven bone density at the distal ends of the bilateral ulnae, radii, femora, and the proximal and distal tibiae and fibulae. The diaphyses of the bilateral femora, tibiae, and fibulae appeared slender, with localized increases in cortical bone density in the bilateral tibiae. Additionally, straightening of the cervical spine curvature and thoracic scoliosis were observed (Figure 3). Developmental assessment using the Griffiths Scales indicated severe delays in gross motor skills, personal-social development, hearing and language, hand-eye coordination, and visual performance.

### 3.4. Genetic Findings

**Figure 4:**
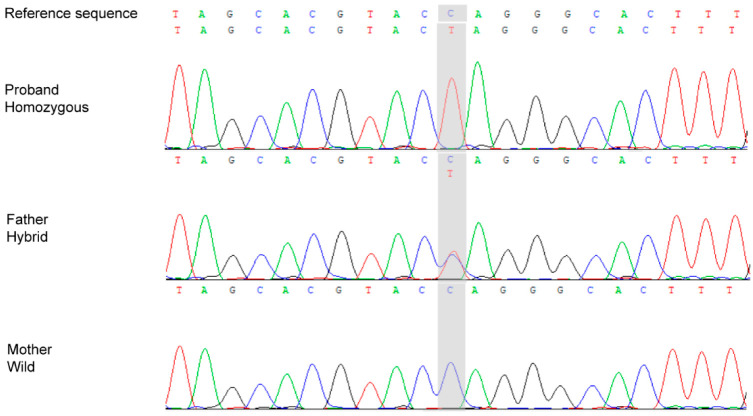
Sanger sequence of the proband and his parents. The chromatograms are displayed in reverse-complement orientation; therefore, the NM_001111035.3:c.311G>A change appears as a C>T signal in the trace. The gray shaded area highlights the exact position of the variant.

WES identified an *ACP5* stop-gain variant (NM_001111035.3:c.311G>A; p.Trp104*) with an apparently homozygous read pattern in the proband (75/75 reads), whereas the father was heterozygous (94/159) and the mother showed a wild-type pattern (0/175). Sanger sequencing confirmed the *ACP5* sequence variant in the proband and his father, while the mother showed no evidence of the sequence variant (Figure 4). The chromatograms in Figure 4 are displayed in reverse-complement orientation; therefore, the NM_001111035.3:c.311G>A change appears as a C>T signal in the trace.

**Figure 5:**
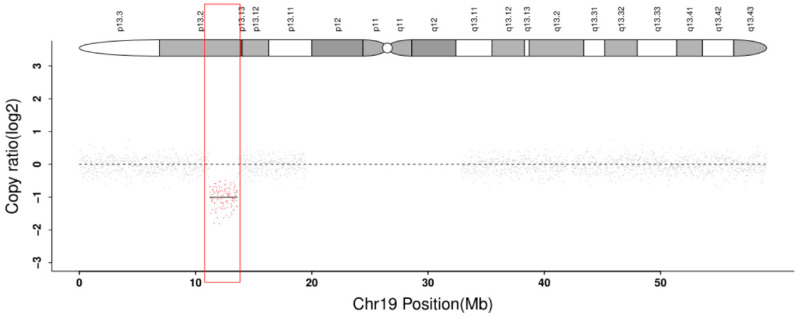
CNV-seq of the proband showing a heterozygous 19p13.2–p13.13 deletion encompassing *ACP5*. The dots represent the log2 copy ratio of individual genomic bins (grey dots indicate normal copy number, while red dots indicate the deleted bins). The shadowed bands on the top chromosome ideogram represent the cytogenetic banding pattern. The red box highlights the exact extent of the deleted region.

Because this segregation pattern was not consistent with a true homozygous sequence variant, the copy-number data were re-evaluated. WES suggested a heterozygous 19p13.2–p13.13 deletion (chr19:11,123,174–13,455,212, 2.332 Mb; GRCh38) involving *ACP5*. CNV-seq subsequently confirmed an overlapping heterozygous 19p13.2–p13.13 deletion in the proband (chr19:11,218,588–13,598,994, 2.380 Mb; GRCh37) (Figure 5). Taken together, these findings indicate biallelic *ACP5* defects consisting of a stop-gain variant on one allele and deletion of the other *ACP5* allele, resulting in an apparent homozygous/hemizygous signal on WES and Sanger sequencing.

According to the original diagnostic report and the ACMG guidelines, c.311G>A (p.Trp104*) was classified as likely pathogenic [11]. PVS1 was applied because this is a nonsense variant, and PM2 was applied because the variant was not recorded in the population databases used in the diagnostic report. No additional phenotype-correlated variants sufficient to explain the patient’s presentation were identified.

### 3.5. Clinical Management and Follow-Up

The patient was treated with immunoglobulins and prednisone acetate, which stabilized hemoglobin and platelet levels. He experienced recurrent infections before the age of two, and each episode required intensive anti-infective therapy to achieve remission. For seizure, he received adrenocorticotropic hormone, prednisone acetate, topiramate, sodium valproate, and levetiracetam sequentially, with satisfactory seizure control. Despite three years of rehabilitation, substantial neurodevelopmental delay persisted on follow-up Griffiths assessments.

## 4. Discussion

SPENCDI is a rare skeletal dysplasia characterized by significant clinical and genetic heterogeneity. The clinical manifestations typically involve metaphyseal and vertebral abnormalities [12]. In the present study, the patient presented with subtle skeletal abnormalities, including a barrel-shaped chest, scoliosis, and varus deformities in both feet. Metaphyseal lesions were not evident until later stages when comprehensive X-ray examinations were conducted. We reviewed previously reported cases of SPENCDI associated with *ACP5* variants (Table 2). Macroscopic skeletal deformities were less common; however, radiographic evaluations revealed metaphyseal or spinal involvement in nearly all patients. Previous studies have primarily described cases with metaphyseal dysplasia and platyspondyly. In contrast, our patient exhibited only metaphyseal dysplasia without platyspondyly, which aligns with the findings reported by Romano et al. and Girschick et al. [13,14]. Notably, the patient also presented extra-osseous manifestations, including recurrent infections and bicytopenia, which are well-documented characteristics of SPENCDI. Furthermore, these extra-osseous symptoms emerged significantly earlier than the skeletal abnormalities. While skeletal changes in SPENCDI typically become apparent after the age of five [15], this patient exhibited recurrent infections and bicytopenia prior to the age of two [16], highlighting an atypical and early onset of extra-osseous symptoms.

**Table 2:** Clinical manifestations and reported JAK inhibitor use in published *ACP5*-related SPENCDI reports.

*ACP5* Variants	Age at Presentation	N	Neurological	Skeletal	Short Stature	Immunodeficiency	Autoimmune	JAK Inhibitor Treatment	Ref.
p.Ser258Trpfs*39	2 m	1	Developmental delay; intracranial calcification	Platyspondyly; metaphyseal lesions	Yes	Recurrent infections	SLE	None	[16]
Homozygous deletion (the specific variant type was unclear)	3 y	1	Intracranial calcification	Platyspondyly; enchondromatous non-ossifying metaphyseal lesions	Yes	None	Thrombotic thrombocytopenic purpura; membranous nephropathy	Baricitinib: long-term remission was achieved	[17]
p.Gly215Arg Het./p.Leu247Pro Het.; p.Ser267Leufs*20 Het./p.Gly239Asp Het.	4 y, 12 y	2	Developmental delay (1/2); headache (1/2); extrapyramidal symptoms (2/2); middle cerebral artery occlusion (1/2); intracranial calcification (2/2); leukodystrophy (2/2)	Platyspondyly + metaphyseal dysplasia (2/2)	1/2	None	Systemic inflammation (1/2)	Tofacitinib (2/2): the patient with a headache experienced symptom relief, while another patient with systemic inflammation had poor disease control	[18]
p.Met264Lys; p.Met264Lys Het./p.Ile211Thr Het.	2 y–6 y	4	Spasticity (1/4); developmental delay (2/4); intracranial calcification (2/4)	Metaphyseal and vertebral dysplasia (4/4); genu valgum (1/4)	1/4	Recurrent infections (2/4); humoral immunodeficiency (2/4)	autoimmune hypothyroidism (1/4); thrombocytopenia (1/4); autoimmune hepatitis (2/4); SLE (1/4); hypocomplementemia (1/4)	Ruxolitinib (2/4): one patient exhibited a partial response due to a relapse of cytopenia, while another achieved an adequate response	[19]
p.Gly109Arg Het./p.Arg176* Het.; p.Gln245* Het./p.Gly204Asp Het.	3 y, 4 y	2	Developmental delay (1/2); intracranial calcification (2/2)	Platyspondyly + metaphyseal dysplasia (2/2)	2/2	None	Evans syndrome (2/2)	Ruxolitinib (2/2): the resolution of cytopenias; the increase in energy, academic performance and general wellbeing	[20]
p.Ex4_7del	13 m	1	Neurological regression; developmental delay; dystonic posturing; possible intracranial calcification	Irregularities in long bone metaphysis and irregular vertebral segments contours without significant platyspondyly	Yes	None	Neutropenia; thrombocytopenia	Baricitinib: IFN score decreased; but the benefit on the neurological aspects is less clear	[13]
p.Lys52Thr	35 y	1	Seizures, myoclonus (due to intracranial infection)	Platyspondyly, enchondromatous changes in bone	Yes	Recurrent opportunistic infection; deep neutropenia, CD4 lymphopenia	SLE, autoimmune myelofibrosis	Baricitinib: the reduction in fatigue levels; mean white blood cell counts and neutrophil counts have increased	[21]
p.Gln184Serfs*28	1 y	1	Spastic diplegia; intracranial calcification	None	Yes	None	Evans syndrome	None	[22]
p.Asp203Ala	2.7 y	1	Seizures; spasticity; developmental delay; neurological regression	Platyspondyly; radiolucent lesions in vertebral bodies; varus abnormality of both feet	Yes	None	Autoimmune hemolytic anemia	None	[15]
p.Thr44Met Het./p.Lys272Glnfs*14 Het.	6 m	1	Spasticity; developmental delay; seizures; cerebral vascular disease	Discrete metaphyseal irregularities	Yes	Recurrent severe infection; reduced number of CD4+, B and natural killer cells;	Autoimmune hemolytic anemia; thrombocytopenia; polyarthritis; anti-phospholipid syndrome; hepatitis; nephritis	None	[14]
p.Gly215Arg; p.Lys52Thr; p.Gln223X	3–6 y	3	Spastic diplegia (2/3); intracranial calcification (2/2, 1 NA)	Platyspondyly + metaphyseal lesions (3/3)	3/3	None	Thrombocytopenia + neutropenia (1/3); thrombocytopenia (1/3); autoimmune hemolytic anemia (1/3)	None	[9]
p.Lys52Thr	2 y, 19 y	2	Spastic paraparesis (2/2); mild cognitive impairment (2/2); intracranial calcification (1/2)	Platyspondyly + metaphyseal changes (2/2)	2/2	None	SLE (2/2)	None	[23]
p.Arg46Trp Het./p.Val150Glu Het.	3 y, 9 y	2	Moyamoya syndrome (2/2)	Platyspondyly + metaphyseal dysplasia (2/2)	None	None	SLE (2/2)	None	[24]
p.Ser210Phe; p.Arg176Ter; p.Gln248ProfsTer3; p.Gly259Arg	4 m–1.7 y	5	Spasticity (4/5); developmental delay (4/5); epilepsy (1/5); intracranial calcification (4/5)	Platyspondyly + metaphyseal dysplasia (5/5); scoliosis (1/5); thoracic kyphosis (1/5)	4/5	Cellular immunodeficiency (3/5) or combined immunodeficiency (1/5)	All patients presented mild autoimmune manifestations	None	[25]
p.Ser258TrpfsTer39	3 m–13 y	9	Spastic paraparesis (5/9); developmental delay (5/9); seizures (1/9); intracranial calcifications (5/6, 3 NA)	Platyspondyly/metaphyseal dysplasia (9/9); scoliosis (1/9); hip dislocation (1/9)	9/9	Recurrent infections (5/9); All of them had normal total lymphocyte counts	Autoimmune hemolytic anemia (5/9), autoimmune thrombocytopenia (3/9)	None	[26]
17 types ^[2]^	Birth–15 y	26	Spasticity (11/25, 1 NA); developmental delay (7/25, 1 NA); intracranial calcification (9/14, 12 NA); others: ataxia, seizures, psychosis and painful multifocal neuropathy	Platyspondyly + metaphyseal dysplasia (23/25, 1 NA); platyspondyly/metaphyseal dysplasia (2/25, 1 NA); short distal phalanges (2/25, 1 NA); kyphosis and pectus carinatum (1/25, 1 NA)	24/25 (1 NA)	Recurrent infection (5/25, 1 NA)	Autoimmune manifestations were present in 22 patients	None	[7] ^[1]^
10 types ^[2]^	10 m–16 y	14	Spasticity (3/13, 1 NA); developmental delay (3/13, 1 NA); intracranial calcification (6/7, 7 NA);	Platyspondyly + metaphyseal dysplasia (14/14); scoliosis (1/14)	14/14	None	Autoimmune manifestations were present in 12 patients (1 NA)	None	[3] ^[3]^

Abbreviations: N, number of patients in the corresponding report; JAK, Janus kinase; Ref., reference; m, months; y, years; SLE, systemic lupus erythematosus; IFN, interferon; NA, not applicable. Data are presented at the report level rather than as a pooled analysis. Fractions such as 1/2 indicate the number of patients with the specified feature within the corresponding report. ^[1]^ This study includes 10 previously reported cases. ^[2]^ Detailed variant sites are provided in Table 3. ^[3]^ This study includes 4 previously reported cases.

**Table 3:** Reported *ACP5* variant sites summarized from References [3] and [7].

*ACP5* Variants	Ref.
p.Lys52Thr	[3]
p.Tyr74X	
p.Gly109Arg	
p.Gly109Arg Het./p.Tyr278del Het.	
p.Leu201Pro	
p.Tyr206X	
p.Gly215Arg	
p.Asn262His	
p.Met264Lys	
p.Ser267X	
p.Ex4_7 del	[7]
p.Ex5_7 del	
p.Ser258Trpfs*39	
p.Thr44Met Het./p.Cys238Arg Het.	
p.Lys52Thr	
p.Lys52Thr Hom./p.Met264Val Het.	
p.Thr89Ile	
p.Gly109Arg	
p.Gly109Arg Het./p.Cys238Arg Het.	
Gln120Arg	
p.Tyr123X Het./p.Asp241Asn Het.	
p.Gly215Arg	
p.Gln223X	
p.His242Arg	
p.Met264Val	
p.Val274Ala	
p.? Hom.	

Extra-osseous manifestations of SPENCDI exhibit considerable heterogeneity, primarily involving immune and neurological abnormalities. A broad spectrum of autoimmune manifestations has been observed in SPENCDI, including hepatitis, nephritis, pancreatitis, rheumatic fever, pancytopenia, hemolytic anemia, autoimmune thrombocytopenia, hypothyroidism, polymyositis, vasculitis, Sjögren’s syndrome, vitiligo, Moyamoya syndrome, systemic lupus erythematosus, juvenile rheumatoid arthritis, Raynaud’s disease, scleroderma, celiac disease and other autoimmune disorders [7,14,24,25]. Although our patient did not fulfill diagnostic criteria for a specific autoimmune disorder, he had marked bicytopenia together with recurrent severe infections and impaired T- and NK-cell cytotoxicity. This finding aligns with the results reported by Briggs et al., who identified recurrent infections in 5 out of 26 SPENCDI patients and documented reduced T-, B-, and NK-cell counts in two of the cases [7]. These findings support the notion that immunodeficiency is a key feature of *ACP5*-related disease, contributing to its clinical complexity.

Neurological involvement is also common in SPENCDI and may include seizures, spasticity, global developmental delay, and intracranial calcifications [14]. In our patient, seizures, global developmental delay, basal ganglia calcifications, and structural brain abnormalities were all present. Published reports indicate that intracranial calcification is a recurrent neuroimaging finding in *ACP5*-related disease; however, neurological severity does not appear to correlate perfectly with the extent of calcification. Some patients with substantial neurological impairment may have limited calcification, whereas others with calcification may be relatively asymptomatic. These observations suggest that neurological dysfunction in SPENCDI is not explained solely by intracranial calcification. As demonstrated by Girschick et al., elevated type I interferon (IFN-I) levels in the cerebrospinal fluid of these patients suggest that overactivation of the immune system constitutes an additional pathological mechanism [14].

Our literature review also supports the clinical importance of targeted skeletal imaging. Across published case reports and case series, metaphyseal and/or vertebral abnormalities were repeatedly described, although obvious skeletal deformities were often absent or subtle. Because some reports may contain overlapping patients and clinical reporting was not uniform across studies, we did not calculate pooled percentages or a unique-patient total. Nevertheless, the overall literature consistently indicates that skeletal involvement, immune manifestations, growth abnormalities, and neurological findings are central features of *ACP5*-related SPENCDI (Table 2). Therefore, SPENCDI should be considered in children with unexplained immune dysfunction, growth disturbance, and developmental or neurological abnormalities, even when overt skeletal deformities are not initially evident.

SPENCDI has been recognized as a type I interferonopathy characterized by persistent hyperactivation of the IFN-I signaling pathway through the Janus kinase-signal transducer and activator of transcription (JAK-STAT) cascade. Research suggests that JAK inhibitors can partially suppress IFN-I receptor signaling and may help attenuate disease activity in type I interferonopathies [27,28]. Aicardi-Goutières syndrome, another type I interferonopathy with partial phenotypic overlap with SPENCDI, has been more extensively studied in this context, and the accumulated evidence supports the potential utility of JAK inhibition in refractory immune-mediated disease. In comparison, published experience with JAK inhibitor therapy in SPENCDI remains limited and warrants further investigation.

In our structured narrative review of the published literature, we summarized the reported therapeutic use of JAK inhibitors in patients with *ACP5*-related SPENCDI (Table 2). Available reports suggest that these agents may mitigate excessive immune activation and improve autoimmune manifestations in some patients [13,17,18,19,20,21]. As discussed above, neurological manifestations in SPENCDI may also be related, at least in part, to immune dysfunction [7,14]. However, whether JAK inhibitors can meaningfully improve neurological outcomes in SPENCDI remains uncertain. In Romano et al.’s study, baricitinib treatment was not associated with clear improvement in neurological manifestations, whereas in Gernez et al.’s study, two patients showed improved neurological outcomes after ruxolitinib therapy. This discrepancy may be attributable to variations in cerebrospinal fluid concentrations of JAK inhibitors [13,20]. Further studies are therefore needed to clarify the potential role of JAK inhibitors in neurological manifestations of SPENCDI. Furthermore, infections have emerged as a predominant concern among the reported adverse events associated with JAK inhibitors. Prior to initiating JAK inhibitor therapy for SPENCDI, a comprehensive immunological evaluation is strongly recommended, particularly given the potential for exacerbating infection risks in patients with immunodeficiency.

SPENCDI is a rare autosomal recessive disorder caused by homozygous or compound heterozygous variants in the *ACP5*. *ACP5*, located on chromosome 19p13, encodes TRAP, an enzyme important for the function of osteoclasts and immune cells. Osteopontin (OPN), a major substrate of TRAP, plays a central role in skeletal and immune homeostasis. Previous studies have shown that loss of *ACP5* function is associated with impaired TRAP activity and abnormal OPN phosphorylation, which in turn has been linked to skeletal dysplasia, brain calcifications, and immune dysregulation. Extracellularly, over-phosphorylated OPN contributes to skeletal dysplasia and brain calcifications associated with neurological dysfunction. Intracellularly, hyper-phosphorylated OPN may enhance IFNα production, disrupting immune regulation and triggering autoimmune diseases. Hyper-phosphorylated OPN may contribute to functional impairment in T/NK cells, suggesting a possible link between *ACP5* deficiency and immunodeficiency [2,3,4]. Our patient’s immunological testing demonstrated markedly reduced cytotoxic activity of T cells and NK cells, potentially underpinning the recurrent infections. Pathogenic biallelic *ACP5* variants are expected to reduce or abolish TRAP activity on the basis of previously reported *ACP5* disease biology. In turn, TRAP deficiency may contribute to immune dysregulation and skeletal abnormalities. Furthermore, the hyperphosphorylation of OPN, which induces brain tissue calcification, together with the hyperactivation of IFNα, may constitute a common mechanism underlying neurological abnormalities [7,14,29]. However, TRAP activity, IFN-I signature, and related in vitro functional assays were not directly assessed in the present patient. Therefore, mechanistic inferences regarding TRAP dysfunction, OPN-related effects, and IFN-I pathway activation in this case should be interpreted cautiously and are based on previously established *ACP5* disease biology, the predicted loss-of-function nature of the variant, and the overall consistency of the phenotype with previously reported SPENCDI cases, rather than on direct functional confirmation in this patient. Across the reports included in our review, approximately 39 distinct *ACP5* variants were identified, with no clear genotype–phenotype correlation identified.

This study has several limitations. First, direct functional validation was not available, including TRAP enzyme activity assays, IFN-I signature assessment, or other in vitro studies using patient-derived cells. Second, the literature component was designed as a narrative review based on a limited number of heterogeneous published case reports and case series, some of which may include overlapping patients. Therefore, the published data were not pooled quantitatively, and conclusions regarding the efficacy and safety of JAK inhibitors should be interpreted cautiously.

## 5. Conclusions

We describe an infant with *ACP5*-related SPENCDI caused by biallelic *ACP5* defects, including a stop-gain variant and an overlapping deletion involving the second *ACP5* allele. This case expands the clinical and molecular spectrum of *ACP5*-related SPENCDI. The case is also notable for the early prominence of immunological and neurological manifestations before definite skeletal abnormalities were recognized radiographically. In addition, our structured narrative review summarizes the currently reported experience with JAK inhibitor therapy. Early genetic evaluation and targeted skeletal imaging may facilitate timely diagnosis in children with unexplained immune dysfunction, developmental delay, and suggestive neuroimaging findings. Future studies including direct functional evaluation, such as TRAP activity assays, IFN-I signature assessment, or other in vitro validation approaches, will be important to further clarify the biological consequences of *ACP5* defects.

## Figures and Tables

**Table 1:** Clinical manifestations and treatment outcomes in the patient.

Category	Age at Onset	Manifestations	Treatment
Neurological	Birth	Seizures, muscle tone abnormalities, basal ganglia calcification, brain dysplasia	Adrenocorticotropic hormone/prednisone acetate/topiramate/sodium valproate/levetiracetam; seizures were well-controlled
Skeletal	Birth	Barrel-shaped chest, scoliosis, varus deformities, and dorsiflexion of the first metatarsophalangeal joints in both feet; metaphyseal lesions	None
Autoimmune	Birth	Thrombocytopenia, anemia	Immunoglobulins + prednisone acetate; blood cell counts normalized
Immunodeficiency	1. Birth 2. 3 m 3. 1 y 4. 1.9 y 5. 2 y	1. Early-onset sepsis and neonatal necrotizing enterocolitis 2. Severe pneumonia 3. Pneumonia 4. Severe pneumonia and enteritis 5. Chronic pneumonia	1. Cefoperazone-Sulbactam/Meropenem 2. Amoxicillin-Clavulanate/Vancomycin+ Meropenem 3. Amoxicillin-Clavulanate 4. Cefoperazone-Sulbactam/Piperacillin-Tazobactam 5. Piperacillin-Tazobactam; symptoms were alleviated following anti-infective treatment
Growth and development	6 m (gross motor)	Developmental delay; height is normal; weight was normal until age 2 then declined, with a Z-score as low as −1.8	Despite adjustments in nutrition and rehabilitation training, there was no significant improvement in weight and development

Abbreviations: m, months; y, years.

## Data Availability

All data generated during this study are included in this published article.
